# One probe fits all: a highly customizable modular RNA *in situ* hybridization platform expanding the application of SABER DNA probes

**DOI:** 10.1242/dev.204775

**Published:** 2025-06-02

**Authors:** Kirill Ustyantsev, Mattia Stranges, Filippo Giovanni Volpe, Jan Freark de Boer, Folkert Kuipers, Stijn Mouton, Eugene Berezikov

**Affiliations:** ^1^ European Research Institute for the Biology of Ageing, University Medical Center Groningen, University of Groningen, Groningen 9700AD, The Netherlands; ^2^ Department of Biological, Geological, and Environmental Sciences, Alma Mater Studiorum Università di Bologna, Bologna 40126, Italy; ^3^ Department of Pediatrics, University Medical Center Groningen, University of Groningen, Groningen, 9713 GZ, The Netherlands; ^4^ Department of Laboratory Medicine, University Medical Center Groningen, University of Groningen, Groningen, 9713 GZ, The Netherlands

**Keywords:** RNA *in situ* hybridization, RNA FISH, HCR, TSA, SABER, *Macrostomum lignano*

## Abstract

RNA *in situ* hybridization (ISH) is a key method for visualizing gene expression patterns in complex samples. ISH is indispensable for research in development, disease, gene function, and validation of novel cell types identified using single-cell sequencing methods. In non-mammalian models lacking accessibility to a broad spectrum of antibodies, ISH remains a major research tool. Available ISH protocols require different custom hybridization probe types, designs and/or proprietary signal detection chemistry. This makes it difficult for a beginner to navigate and increases research costs when multiple methods need to be applied. Here, we describe OneSABER, a unified open platform connecting commonly used canonical and recently developed single- and multiplex, colorimetric and fluorescent ISH approaches. OneSABER uses a single type of DNA probes adapted from the signal amplification by exchange reaction (SABER) method. We demonstrate the applications, versatility and efficiency of the OneSABER framework in whole-mount samples of the regenerative flatworms *Macrostomum lignano* and *Schmidtea mediterranea* and formalin-fixed, paraffin-embedded mouse intestinal sections. Comprehensive comparison of the most suitable ISH signal development techniques is discussed.

## Teaser

**Summary:** Demonstration of OneSABER as a highly customizable modular RNA *in situ* hybridization platform expanding the application of SABER DNA probes to allow diverse signal development and amplification techniques to be combined in a unified framework.

## INTRODUCTION

Dynamic changes in spatiotemporal gene expression in different cell types, tissues and organs reflect the complex molecular regulation taking place in an organism in response to internal and external stimuli. Studying these dynamics is crucial for understanding the roles of specific genes in key biological processes, such as embryonic development, homeostasis and regeneration, in both normal and disease conditions. RNA *in situ* hybridization (ISH) and immunohistochemistry methods have allowed researchers to directly detect, measure and visualize transcriptional and post-translational expression patterns of genes within native anatomical and histological contexts, connecting gene expression to the source cell (Hemmati-Brivanlou et al., 1990; Jowett and Lettice, 1994; Tautz and Pfeifle, 1989; Toledano et al., 2012; Young et al., 2020). Owing to the paucity or even complete absence of commercial antibodies for most non-mammalian species (Hadwiger et al., 2010; Peter et al., 2017), ISH remains the main research tool for studying gene expression patterns in non-canonical models. Furthermore, the rapid expansion and broad accessibility of next-generation single-cell sequencing technologies have enabled the discovery of potential novel cell types and their marker genes, which require experimental validation by *in situ* methods (Chari et al., 2021; Fincher et al., 2018; Hulett et al., 2023).

For decades, enzyme-catalyzed reporter deposition assays, such as alkaline phosphatase (AP) colorimetric ISH and horseradish peroxidase (HRP) tyramide signal amplification (TSA) fluorescent ISH (FISH), have been the gold standard for achieving reliable high-signal-over-noise RNA and protein assays in whole-mount preparations (Bobrow et al., 1989; Jowett and Lettice, 1994; Luehrsen et al., 2000; Schmidt et al., 1997; Tautz and Pfeifle, 1989; Young et al., 2020). To date, many different open-access and commercial (F)ISH approaches have been developed, focusing on optimizing the methods' sensitivity (signal amplification), specificity (low off-target signal), resolution (down to subcellular localization), and protocol implementation time, while allowing multiplexing – simultaneous visualization of multiple target molecules in the same sample (Dardani et al., 2022; Goh et al., 2020; Kishi et al., 2019; Schulte et al., 2024; Tao et al., 2023; Wang and Guo, 2021; Wang et al., 2012; Wu et al., 2022; Xia et al., 2019; Young et al., 2020). Excelling in all these aspects of ISH is a big challenge when working with complex, thick and highly autofluorescent whole-mount samples. This leaves many multiplexing-focused tools impractical beyond the levels of cell cultures or thin tissue sections (Schulte et al., 2024). Therefore, there is still room for canonical AP-based and TSA FISH methods, despite their limited multiplexing capabilities and cell-only resolution due to signal diffusion (Schulte et al., 2024; Wu et al., 2022). Additionally, the lower entry costs and robustness of canonical techniques make them indispensable for initial research to obtain the ground-truth pattern of gene expression.

Regardless of the ISH method, a new user will face the fact that every approach is tailored for specific antisense target-binding probes, which mainly fit only one of the methods. This thus requires either locking-in to a particular technique or substantial resource investments to accommodate several methods in one project. Additionally, when using commercial solutions, the exact probes' design can sometimes be hidden from the end user, obscuring the research data and hindering troubleshooting and the independent design of the study. Challenged by these issues, we sought open, non-commercial alternatives that allow us to combine the benefits of canonical ISH approaches and modern semi-proprietary methods. We were inspired by recent advancements in the generation of long single-stranded (ssDNA) probes from short ssDNA oligonucleotides through primer exchange reaction (Kishi et al., 2018, 2019), which were implemented in a family of separate signal amplification by exchange reaction (SABER) protocols, including the original SABER FISH (Kishi et al., 2019), Immuno-SABER (Saka et al., 2019), and the most recent and sensitive pSABER method utilizing the TSA assay (Attar et al., 2025). We reasoned that such probes can be simply and universally combined with canonical colorimetric anti-hapten antibody-mediated AP- and HRP-based ISH and FISH assays, as well as with the more recent enzyme-free one-step hybridization chain reaction (HCR) multiplex FISH (Choi et al., 2014, 2018; Schulte et al., 2024). Here, we demonstrate a ‘one probe fits all’ approach, OneSABER, in which a once-ordered set of generic user-defined short ssDNA oligonucleotides forms a platform for adaptable RNA (F)ISH signal amplification strength via SABER concatemers with customizable length. OneSABER is suitable for all the commonly used detection methods.

The free-living marine flatworm *Macrostomum lignano* is a relatively new invertebrate model for studying regeneration, *in vivo* stem cell potency and differentiation, aging, genome duplication and karyotype evolution, sexual selection, and many more biological questions (De Miguel-Bonet et al., 2018; Egger et al., 2006; Giannakara et al., 2016; Grudniewska et al., 2016; Mouton et al., 2018; Patlar et al., 2020; Pfister et al., 2008; Santhosh et al., 2024; Ustyantsev and Berezikov, 2021; Ustyantsev et al., 2021a; Wudarski et al., 2020; Wunderer et al., 2019; Zadesenets et al., 2017, 2023). Amenability to crucial molecular biology techniques, such as RNA interference and, most importantly, transgenesis, combined with convenient laboratory culturing conditions and features such as a transparent body, make *M. lignano* a powerful and increasingly popular model organism (Hall et al., 2024; Mouton et al., 2024; Ustyantsev et al., 2021b; Wudarski et al., 2017, 2020). However, RNA ISH in *M. lignano* has been limited mostly to canonical AP-based colorimetric assays using long hapten-labeled antisense RNA probes (Grudniewska et al., 2016, 2018; Kuales et al., 2011; Pfister et al., 2007, 2008; Wudarski et al., 2017; Wunderer et al., 2019; Zhou et al., 2015), hindering the experimental potential of this flatworm. As a proof-of-concept implementation of the OneSABER ISH platform, we demonstrate its utility for RNA ISH and multiplex TSA and HCR FISH in whole-mount *M. lignano* samples*.* Additionally, we provide a detailed protocol for the ‘one probe fits all’ SABER whole-mount ISH in *M. lignano* with liquid-exchange mini-columns (Irvine, 2007) to minimize sample losses and shorten the overall experimental time. Importantly, the method and protocol are easily adaptable to other models, which we demonstrate with whole-mount planarians and fixed, paraffin-embedded mouse tissue sections.

## RESULTS AND DISCUSSION

### Overview of the OneSABER platform principles and its components

**Fig. 1.**
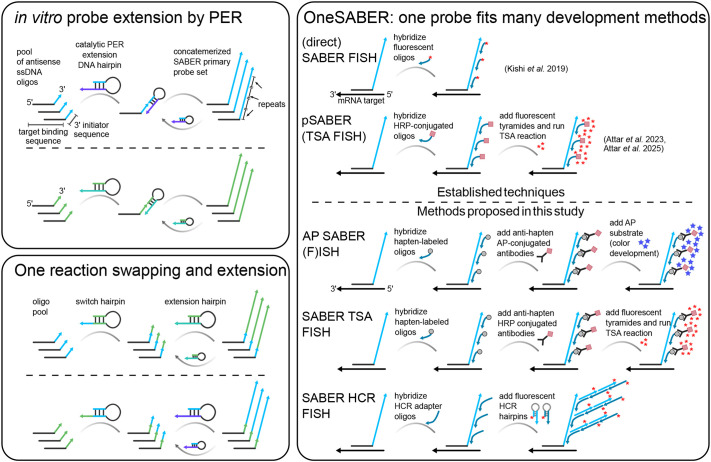
**Principal operation scheme of the OneSABER mRNA *in situ* hybridization platform.** Two key features lie at the heart of the ‘one probe fits all’ concept: (1) using the same once-ordered pools of antisense ssDNA oligonucleotides to generate different concatemerized 3′ end sequences of various lengths through a primer exchange reaction (PER) amplification cycle (Kishi et al., 2018, 2019) for binding universal secondary probes (left); and (2) adaptability of the generated elongated probes to different signal development and amplification methods through universal secondary probes (right). Different colors of upright arrows and hairpins indicate the different nucleotide sequences of the corresponding concatemers and extensions/switch PER hairpins. Fluorophores are labeled as red asterisks. Haptens are indicated by gray circles. AP, alkaline phosphatase; HCR, hybridization chain reaction (Choi et al., 2018); HRP, horseradish peroxidase; SABER, signal amplification by exchange reaction (Kishi et al., 2019); TSA, tyramide signal amplification. See Figs S1, S2 and S6B for additional details on the probes/fluorophores switching and multiplexing. A detailed comparison of different OneSABER signal development methods, their most suitable experimental application, and associated costs is given in Tables S4-S6.

We developed the ‘one probe fits all’ OneSABER platform as a modular approach, stemming from classical ISH protocols and several recently developed methods. The aim is to provide a unified, versatile and fully user-controlled ISH experimental design. The principal operation of OneSABER is illustrated in Fig. 1. At the heart of the platform is a pool of 15-30 custom user-defined short (∼35-45 nt) ssDNA oligonucleotides complementary to an RNA target. The number of probes depends on the target RNA length, its expression strength, chosen signal development method, desired resolution, and available budget. The target length defines a physical limit for the maximum number of probes while, at the same time, weakly expressed genes may require more probes for the same signal development method than targets with higher expression levels (see supplementary Materials and Methods, Section S1). Each probe is ordered with a specific 9 nt 3′ initiator sequence and is extended *in vitro* through a primer exchange reaction (PER) (Kishi et al., 2018) (supplementary Materials and Methods, Section S1). PER relies on a catalytic DNA hairpin combined with a strand-displacing polymerase and competitive branch migration to repeatedly add the same sequence to the 3′ end of ssDNA primers, thus generating long concatemerized probes. The length of the extension is controlled by simply changing the reaction time and underlies the signal amplification strength (Kishi et al., 2019). The concatemers serve as universal landing-pad sequences for binding short (20 nt) secondary oligonucleotide ssDNA probes/adapters, which are modified according to the chosen signal development method. Secondary probes labeled with hapten molecules such as digoxigenin (DIG) and fluorescein (FITC/FAM) allow the application of standard enzyme-catalyzed reporter deposition assays, which are mediated by anti-hapten antibodies conjugated with AP or HRP for colorimetric and TSA fluorescent signal detection, respectively (Harper et al., 1997; King and Newmark, 2013; Paganos et al., 2022). Adding 5′ overhangs with HCR initiator sequences to the secondary probes converts them into adapters for fluorescent signal detection by HCR amplification (Choi et al., 2018; Wu et al., 2022) (Table S1). By changing the initiator sequence on the adapter probes, the same primary SABER probe pool can be targeted by different pairs of fluorescent HCR amplifiers, thereby switching the detection channel (Figs S1, S6B). Multiplexing is achieved by creating sets of primary SABER probes with different 3′ concatemer sequences, which, in turn, are specifically recognized by secondary probes with either different haptens (DIG, FAM or dinitrophenol) or respective SABER-to-HCR adapters (Fig. S2, Table S1). Importantly, PER allows straightforward, single-step switching of concatemer sequences *in vitro* (Kishi et al., 2019), eliminating the need for ordering new sets of primary probe oligonucleotides (Fig. 1).

Apart from SABER FISH (Kishi et al., 2019), the OneSABER platform has been inspired by several other studies. The idea of using haptenized secondary probes with subsequent antibody-mediated AP colorimetric substrate deposition came from a commercial BaseScope™ branched ISH/FISH protocol (Wang et al., 2019), while our SABER-to-HCR adapters are the result of rethinking of the π-FISH (Tao et al., 2023) and Yn-situ (Wu et al., 2022) adapter and pre-amplifier probes. Antibody-mediated SABER TSA FISH is a modification of the peroxidase pSABER protocol published by the developers of SABER FISH (Attar et al., 2025), where they showed substantial signal improvement when using directly HRP-conjugated secondary probes followed by TSA compared to the original protocol (Kishi et al., 2019).

### Colorimetric OneSABER *in situ* hybridization in *M. lignano*

In *M. lignano*, apart from a very recent HCR application (Hall et al., 2024), only the results of traditional ISH/FISH assays using *in vitro*-transcribed, long antisense RNA probes with hapten-modified uridine bases have been reported (Grudniewska et al., 2018, 2016; Kuales et al., 2011; Lengerer et al., 2018; Mulder et al., 2010; Pfister et al., 2007, 2008; Wudarski et al., 2017; Wunderer et al., 2019; Zhou et al., 2015). Compared to these RNA probes, short synthetic DNA probes provide several advantages, including user-controlled design of the most specific on-target sites, the possibility of targeting short RNA transcripts (Tao et al., 2023), low hands-on processing time, and higher probe stability. We designed seven sets of 15-31 ssDNA probes targeting genes expressed in somatic tissues of the worm (neuronal *syt11*, intestinal *apob*, muscular *tnnt2*), testes (*boll* and *sperm1*), and proliferative cells, including somatic stem cells and germline cells (*piwi* and *pcna*) (Table S2). The probe sets were concatemerized by the PER method to an approximate length of 170-300 nt (Table S3) and used for ISH experiments in *M. lignano* to test different signal development conditions. Our *M. lignano* ISH protocol contains several important modifications compared to previously published methods (Pfister et al., 2007). From the planarian and zebrafish protocols, we used combined fixation and permeabilization by adding acetic acid to the formaldehyde fixative, and we added a permeabilization step with hydrogen peroxide (Fernández and Fuentes, 2013; Gaetano and King, 2023; King and Newmark, 2013; Lauter et al., 2011). Hybridization buffers, wash solutions and hybridization temperatures were adapted to match the requirements of the short binding sequences of the primary and secondary SABER probes. Finally, we found that the use of liquid-exchange mini-columns (Irvine, 2007) instead of microcentrifuge tubes or multiwell plates during pre- and post-hybridization steps can prevent the loss of smaller animals as well as make the exchange of hybridization and wash solutions faster, more convenient and less wasteful (supplementary Materials and Methods, Section S2).

**Fig. 2.**
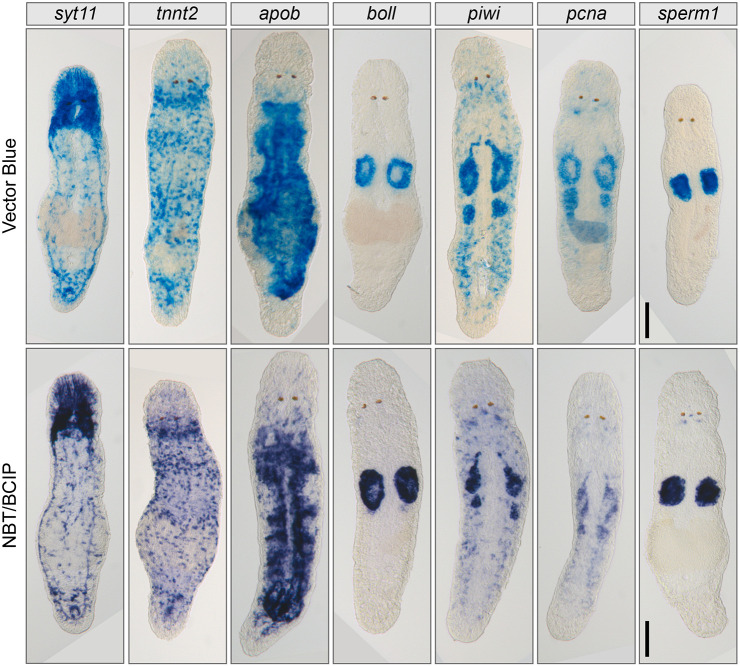
**Example of AP SABER colorimetric signal development on whole-mount samples.** *M. lignano* samples using different AP substrates. Vector Blue can also be viewed by fluorescence microscopy (Fig. S3). NBT/BCIP, nitro-blue tetrazolium chloride/5-bromo-4-chloro-3′-indolyphosphate p-toluidine salt. See Tables S2 and S3 for the development times and the number of probes used for each mRNA target. Scale bars: 50 µm.

First, we tested colorimetric signal development with AP SABER ISH on adult homeostatic (uncut) *M. lignano* worms using two different AP substrates: Vector Blue (Vector Laboratories) and nitro-blue tetrazolium chloride/5-bromo-4-chloro-3′-indolyphosphate p-toluidine (NBT/BCIP) (Fig. 2). Already after 10 min of development, strong and specific signals could be detected for the *boll*, *apob* and *sperm1* genes, whereas for *syt11*, *tnnt* and *piwi* it took approximately 40 min, and almost 2 h for *pcna* (Fig. 2, Table S3). In some cases, however, we noticed recurring non-specific signal around the worms' mouth and pharynx, reminiscent of the position of *M. lignano* secretion cells in the head (Lengerer et al., 2016). This background was independent of the SABER concatemer sequence and probe set. This pattern appeared to be specific to the SABER protocol and differed from the standard ISH background in the gut and/or pharyngeal glands frequently observed with long antisense RNA probes in *M. lignano* (Lengerer et al., 2018; Pfister et al., 2007). In later experiments, we noticed that non-specific signal was completely absent when using younger worms (less than 6 weeks old) and shorter (<500 nt) extended primary probes. ISH patterns of *Syt11* (neuronal), *tnnt2* (muscle) and *pcna* (neoblasts) were obtained for the first time in *M. lignano*, while the patterns of the remaining genes corresponded to those previously published (Kuales et al., 2011; Pfister et al., 2007, 2008; Wudarski et al., 2017). *pcna* expression strongly resembled that of *piwi* (Grudniewska et al., 2016; Pfister et al., 2007), and the *tnnt2* pattern was similar to the previously published pattern of *myh6* (Wudarski et al., 2017). The pattern of *syt11* corresponded to the described anatomy of the *M. lignano* neural system (Hall et al., 2024; Ladurner et al., 1997, 2005; Morris et al., 2007).

Colorimetric single-gene ISHs should serve as ground truth for finding the correct gene expression, estimating the sensitivity and specificity of the ordered probes, and should show whether adjustments to the concatemer length are necessary before proceeding to less-sensitive FISH assays. The samples can be stored indefinitely for archiving in common mounting media. Vector Blue precipitates, such as Fast Blue (Brown and Pearson, 2015; Lauter et al., 2011), could also be visualized fluorescently (Fig. S3) in the near far-red light spectrum and scanned by confocal microscopes without photobleaching by even strong laser powers. Additionally, these fluorescent substrates can be multiplexed with other FISH development assays, such as TSA (Gaetano and King, 2023; Lauter et al., 2011; Schumacher et al., 2014).

### Fluorescent and multiplexed OneSABER *in situ* hybridization in *M. lignano*

On fixed cells and thin tissue sections, the original unbranched version of SABER FISH has shown high flexibility and ease of multiplexing by using directly fluorophore-labeled secondary probes called imagers (Kishi et al., 2019). This approach has been validated in several independent studies (Amamoto et al., 2019; Chrysostomou et al., 2022; Wang et al., 2020). However, when we tried to apply the original direct SABER FISH on whole-mount preparations of *M. lignano*, it resulted in low fluorescence intensity levels. This was even the case for strongly expressed targets such as *syt11*, which had the highest number of ordered probes (Fig. S4, Table S2)*.* For weaker targets, such as *piwi*, the signal was barely detected, even by confocal microscopy (Fig. S4). A lower signal in the absence of amplification through the hybridization of secondary and tertiary branch probes was also acknowledged for the SABER FISH technique (Attar et al., 2025). Branching would make the protocol longer and more complicated, while ordering more primary probes would be costly, and the additional extension of concatemers may compromise their penetration into cells and increase background labeling. As a solution, Attar et al. developed the pSABER (TSA FISH) protocol (Attar et al., 2025). We successfully tested this protocol, which uses secondary probes directly conjugated with HRP enzymes, making it faster than antibody-mediated SABER TSA FISH (Fig. S5A). However, the synthesis of HRP-conjugated oligonucleotides is not commonly offered and is less accessible to most users. It also requires high initial investments, with limited returns (Tables S4 and S5). Furthermore, in the OneSABER platform, it would be more beneficial to use antibody-mediated SABER TSA FISH because it shares the same hapten-modified secondary probes as the AP SABER (F)ISH assay (Fig. 1). Importantly, fluorescent tyramides can be synthesized in-house and have been shown to be efficient for TSA FISH in whole-mount zebrafish and planarian samples (King and Newmark, 2013; Lauter et al., 2011), thus allowing significant potential cost reductions (Tables S4 and S5) compared to commercial alternatives.

**Fig. 3.**
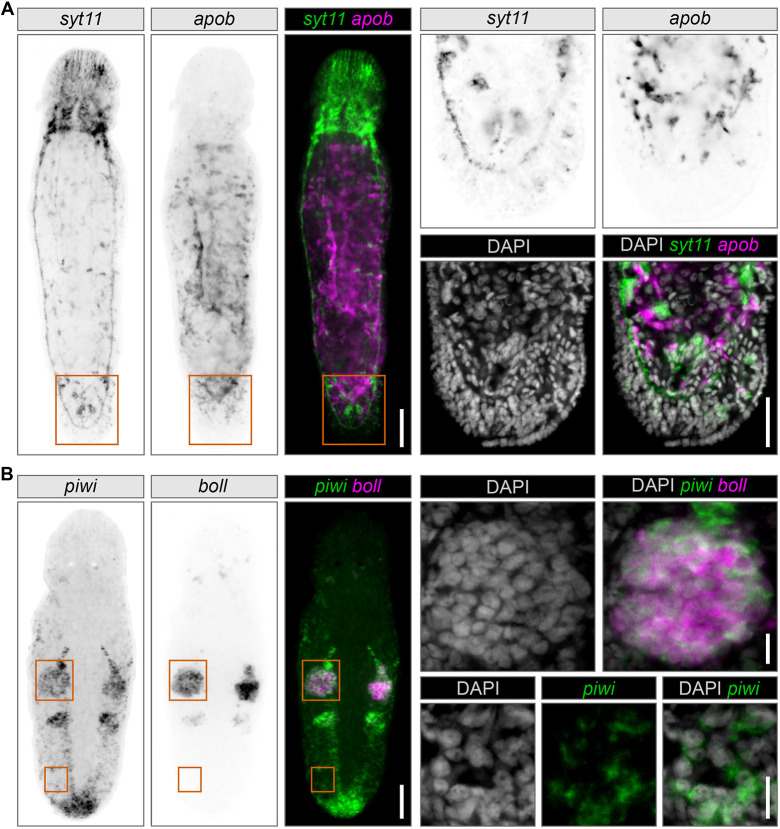
**Example of duplex fluorescent signal development using SABER TSA FISH on whole-mount homeostatic and regenerating *M. lignano* samples.** The three panels on the left are widefield fluorescence microscopy images. Grayscale panels are inverted images of different fluorescent channels provided for unbiased patterns' comparison. Magnified regions are indicated by orange boxes and are shown as panels on the right. Magnifications are the average intensity *z*-projections of several confocal microscopy sections. (A) Expression patterns of a pair of somatic genes, *syt11* (neuronal) and *apob* (intestinal), in a homeostatic worm. Magnifications show the tail plate region. *apob* and s*yt11* were developed with fluorescein (FITC) and CF568 tyramides, respectively. Scale bars: 50 μm (left panels); 25 µm (right panels). (B) Expression patterns for a pair of germline and stem cell genes, *boll* (testes) and *piwi* (germline and stem cells), in a regenerating worm fixed 24 h after tail-plate amputation. Magnifications show the testis and somatic neoblasts. *piwi* and *boll* were developed with FITC and CF647 tyramides, respectively. Scale bars: 50 μm (left panels); 10 µm (right panels).

Therefore, we tested SABER TSA FISH for the simultaneous detection of gene pairs of *syt11* with *apob* in adult uncut worms (Fig. 3A), and *piwi* and *boll* in regenerating worms (Fig. 3B) after tail-plate amputation. Regeneration was employed to boost *piwi* mRNA levels in somatic neoblasts and highlight its expression in the blastema. TSA allows flexible fluorescence channel selection for each gene in a multiplex assay by simply changing the fluorescent tyramides for development (Fig. 3, Fig. S5A). Overall, TSA SABER resulted in high signal-to-noise ratio FISH expression patterns with the whole cell cytoplasm evenly illuminated for both gene pairs.

**Fig. 4.**
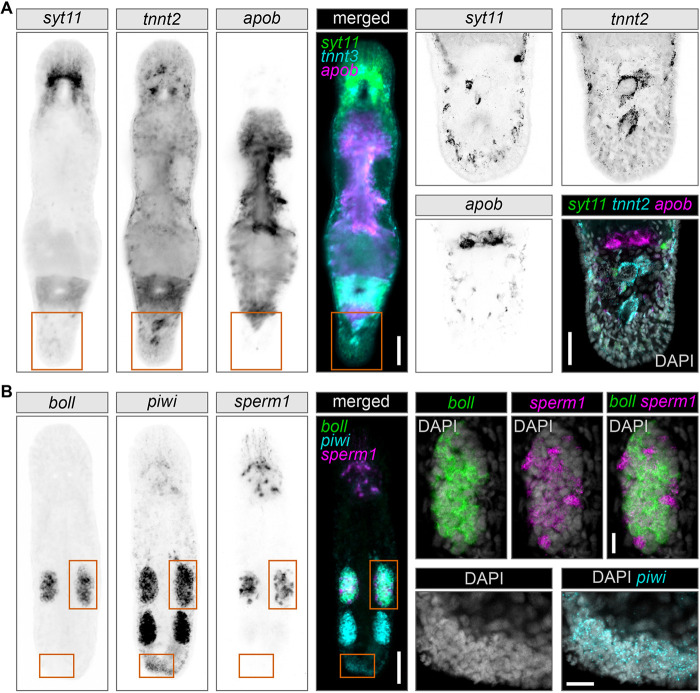
**Example of triple multiplex fluorescent signal development using SABER HCR FISH on whole-mount homeostatic and regenerating *M. lignano* samples.** The four panels on the left are widefield fluorescence microscopy images. Grayscale panels are inverted images of different fluorescent channels provided for unbiased patterns' comparison. Fluorophores from left to right: Alexa Fluor 488, Alexa Fluor 546 and Alexa Fluor 647. Magnified regions are indicated by orange boxes and are shown on the right. Magnifications are average intensity *z*-projections of several confocal microscopy sections. (A) Expression patterns of three somatic genes, *syt11* (neuronal), *tnnt2* (muscular) and *apob* (intestinal), in a homeostatic worm. Magnifications show the tail plate region. Scale bars: 50 μm (left panels); 25 µm (right panels). (B) Expression patterns of three germline and stem cell genes, *boll* (testes), *piwi* (germline and stem cells) and *sperm1* (testes), in a regenerating worm fixed 24 h after tail-plate amputation. Magnifications show the testis and blastema regions. Scale bars: 50 μm (left panels); 10 µm (right panels).

**Fig. 5.**
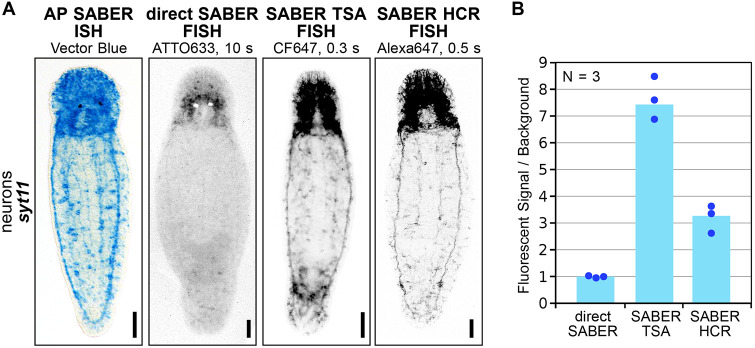
**Comparison of the sensitivity and resolution of different OneSABER development methods for *M. lignano.*** (A) Visual signal-to-noise ratio and resolution of different neuronal structures. The same pool of extended *syt11* probes was used. FISH signals were developed until peak saturation was reached. Widefield brightfield (AP SABER ISH) and fluorescent (other panels) images of representative animals are shown. Grayscale panels are inverted images of different fluorescent channels provided for unbiased patterns' comparison. Colorimetric signal was developed with Vector Blue and the panel is provided as a reference for the FISH methods. FISH signals were developed using different far-red fluorophores, which are indicated below the methods' names. All images were taken using the same objective and magnification, but the exposure times (indicated after the fluorophores) were adjusted to prevent oversaturation of neurons in the worms' posterior. Scale bars: 50 µm. (B) Relative signal over background quantification of *syt11* in *M. lignano* animals developed by the OneSABER FISH methods presented A.

Finally, we tested whether SABER could be used in combination with HCR hairpin amplifiers. We combined probes for *syt11*, *tnnt* and *apob* for SABER HCR FISH in adult homeostatic worms (Fig. 4A) and *boll*, *piwi* and *sperm1* in regenerating animals (Fig. 4B). The samples showed obvious tissue-specific fluorescent signals for each gene, although the patterns appeared as a finer outline than those in SABER TSA FISH (Fig. 3) and appeared as small grainy foci within the cells rather than evenly cytoplasm-diffused TSA fluorescence. As a result, despite the higher intracellular resolution, some fine anatomical structures, such as neural chords (*syt11*) or muscles (*tnnt2*), were less resolved by widefield fluorescence microscopy after SABER HCR FISH than after SABER TSA FISH (Figs 3-5). It is difficult to compare the brightness and strength of different fluorophores, especially in the context of different signal diffusion. When choosing between SABER HCR FISH and SABER TSA FISH, the multiplexing potential must also be considered. Signal development in HCR occurs simultaneously for all channels in a single step. In TSA, it is sequential and takes *N*x more time to develop for *N* different fluorescent signals than in HCR. Therefore, if TSA chemistry and antibodies are not yet available for a user and multiplexing is prioritized over signal strength, then investing in HCR chemistry over TSA might be a good solution (Tables S4 and S5).

The current version of the original HCR FISH protocol relies on short, split-dual primary DNA probes, which have been shown to significantly suppress the background HCR amplification from non-specific probe binding (Choi et al., 2018; Schulte et al., 2024). In principle, the primary split-probe approach is also compatible with the OneSABER platform; however, we have not tested whether it will be beneficial in this setup. In SABER HCR FISH, primary probes are approximately ten times longer, may penetrate less into the sample, and cause unwanted background. However, apart from the previously mentioned inherent non-specific binding to the areas in the worm's head, SABER HCR FISH showed a similar signal specificity to SABER TSA FISH, indicating the viability of our single-probe approach for the whole-mount samples of *M. lignano*.

There are two major advantages of SABER HCR FISH to the original HCR protocol. First, similar to the Yn-situ (Wu et al., 2022) and π-FISH (Tao et al., 2023) methods, longer landing-pad concatemer lengths of SABER probes should provide scalable linear signal pre-amplification and allow the use of fewer probes, thus reducing costs. Second, the same SABER probe can be seamlessly adapted to any HCR hairpin amplifier by simply changing the 5′ initiator sequence of the corresponding SABER-to-HCR adapters. This eliminates the need to order multiple sets of HCR amplifiers labeled with different fluorophores because any SABER probe can be used with any of the HCR hairpins. Ordering only a single set of HCR hairpins substantially reduces the cost of planning complex experiments using fluorescence channel switching. To demonstrate this, we fully swapped the fluorescence channels for the same probes of *sperm1*, *syt11* and *piwi* in another SABER HCR FISH experiment, where we used a mix of annealed HCR amplifiers from previous experiments (Fig. S5B).

### Sensitivity and resolution of different OneSABER signal development methods

When designing an ISH experiment, selecting the signal development approach for the same set of probes can be justified by several criteria: signal intensity and signal-to-noise ratio (sensitivity), signal diffusion (resolution), implementation time, multiplexing capacity, and reagent costs. The latter three criteria are summarized in Tables S4-S6. To illustrate how the choice of the development method affects the peak observed hybridization signal sensitivity and resolution in OneSABER, we repeated the *syt11* experiment using the same batch of probes for all discussed development methods, except for pSABER (TSA FISH) (Fig. 5). Unexpectedly, the HRP-conjugated secondary probes used for pSABER (Tables S1, S4, S5) completely lost their activity after less than a year of storage at 4°C, and we could not repeat the experiment for them. In addition, the implementation of pSABER in our hands had the highest entry cost and price per reaction compared to other (F)ISH methods (Table S4). Perhaps, the issue could be resolved by optimizing its storage conditions. *Syt11* signal was chosen to reflect the diversity of anatomical structures (from relatively narrow neural chords to neuron bodies and ganglions) and because of the high number of probes (31; Table S2), which would allow higher sensitivity for the (direct) SABER method. The colorimetric signal of AP SABER ISH was completed after 45 min of development and was provided as a ground-truth pattern reference for FISH techniques. For the FISH methods, we used the spectrally similar available ‘far-red’ fluorophores ATTO633, CF647 and Alexa Fluor 647 to minimize comparative bias and allow the highest signal over autofluorescence discrimination. Consistent with the results described in the previous sections, the original (direct) SABER FISH showed the lowest signal strength and visual resolution compared with SABER TSA and HCR FISH (Fig. 5, Fig. S4). The strongest SABER TSA FISH signal was, as mentioned before, more diffused and seemingly blurred compared to SABER HCR FISH, which had approximately twice the lower signal level (Fig. 5B). To navigate potential users when planning their experiments, we have summarized the advantages and most suitable applications for each of the OneSABER signal development protocols in Table S6.

### Application of OneSABER in other animal models and sample types

**Fig. 6.**
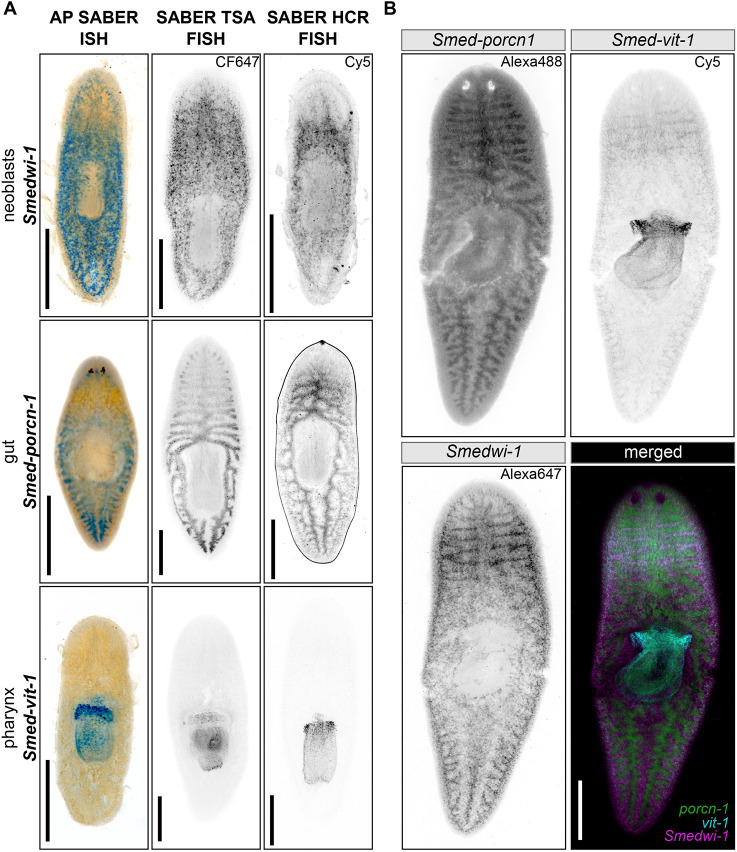
**Example of OneSABER application on whole-mount homeostatic samples of planarian *S. mediterranea.*** All images were obtained using a widefield microscope. Grayscale panels are inverted images of different fluorescent channels provided for unbiased patterns' comparison. Scale bars: 500 µm. Custom-ordered HCR hairpins were used for SABER HCR FISH (Table S1). (A) Single-gene *in situ* hybridizations using the three different development methods (Fig. 1). The development methods and fluorophores used are indicated at the top of the figure. Colorimetric ISH was developed using Vector Blue. Gene names and the corresponding tissues are shown on the left. (B) Multiplex SABER HCR FISH using probes for the three genes shown in A. Originally ordered with p27 3′ initiator sequence, *Smed-vit-1* and *Smedwi-1* probe pools were swapped and extended to p30 and p28 concatemers to enable multiplexing. Fluorophores used are indicated at the top right corners of the panels.

**Fig. 7.**
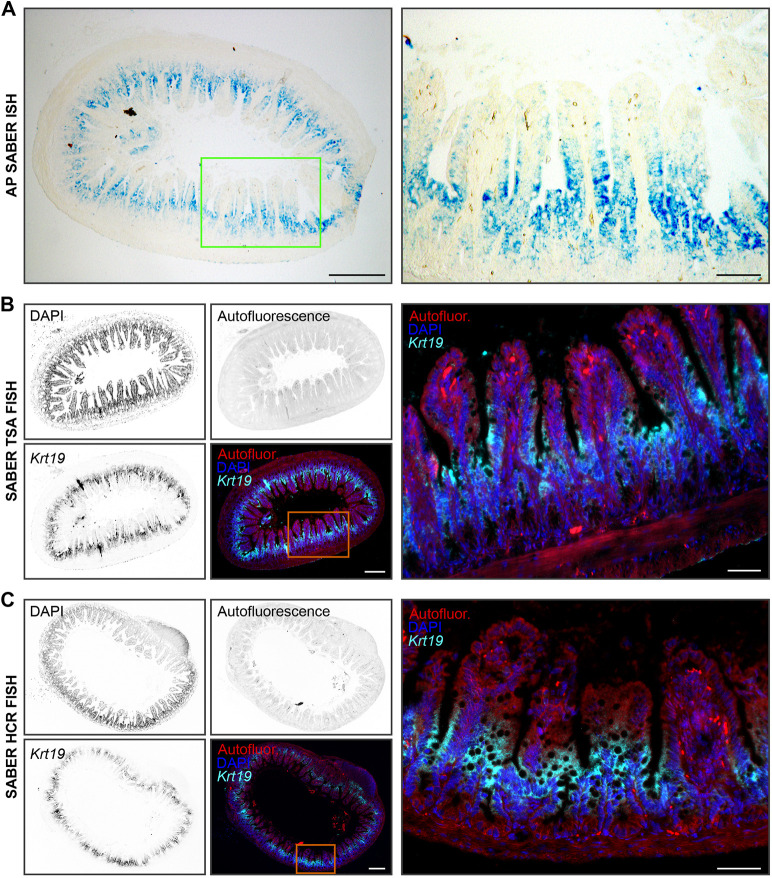
**Example of OneSABER application on mouse small intestine FFPE transverse tissue sections.** Single-gene *in situ* hybridizations of mouse *Krt19* using three different development methods (Fig. 1) are shown. All images were taken using a widefield microscope. Grayscale panels are inverted images of different fluorescent channels provided for unbiased patterns' comparison. The tissue autofluorescence background is from the ‘green’ fluorescent channel. Magnified regions are indicated by green or orange boxes and shown on the right. Scale bars: 200 µm (overview panels; left); 50 µm (magnification panels; right). (A) Colorimetric AP SABER ISH development using Vector Blue substrate. (B) SABER TSA FISH using CF647-labeled ‘far-red’ tyramide. (C) SABER HCR FISH results. Custom-ordered Alexa Fluor 647-labeled ‘far-red’ HCR hairpins were used (Table S1).

To test whether the OneSABER protocols and ‘one probe fits all’ principles could be applied beyond the relatively small whole-mount *M. lignano* samples, we performed a series of ISH experiments on whole-mount specimens of the planarian model *Schmidtea mediterranea* (Fig. 6) and formalin-fixed paraffin-embedded (FFPE) sections of mouse small intestine (Fig. 7). Both sample types introduce specific challenges compared to *M. lignano*. While *S. mediterranea* flatworms are much larger and thicker than *M. lignano*, FFPE sections, although thin and accessible for probe binding, exhibit high autofluorescence and RNA-degradation levels. Notably, FFPE samples remain the main specimen format in clinical and research pathology laboratories (Attar et al., 2025).

For *S. mediterranea*, we designed probes with the same 3′ initiator sequence (p27), targeting genes with published expression patterns from three distinct tissues: somatic stem cells (*Smedwi-1*; 24 probes), gut (*Smed-porcn-1*; 14 probes) and pharynx (*Smed-vit-1*; 20 probes) (Table S2). We first tested three of the most robust signal development techniques (Fig. 5) for each gene separately: AP SABER ISH, SABER TSA and HCR FISH (Fig. 6A). For each gene and developing method, we obtained the same clear patterns as published ones without noticeable off-target binding (Fincher et al., 2018; Gaetano and King, 2023). Next, we performed multiplex FISH using the SABER HCR for all three genes (Fig. 6B). For this, we first swapped the original p27 3′ initiator sequences of *Smedwi-1* and *Smed-vit-1* to p30 and p28 concatemers, respectively, thus demonstrating the universality of the once-ordered oligonucleotide probes (see Fig. 1 and Fig. S6B). Importantly, for all SABER HCR FISH experiments in *Schmidtea*, we used custom-ordered HCR hairpin amplifiers instead of commercial ones from Molecular Instruments, which adds additional flexibility and cost reduction to the experimental design using HCR (Fig. 6, Tables S2, S4-S5).

For mouse FFPE small intestine sections, we designed probes targeting the mRNA of the cytokeratin 19 gene (*Krt19*; 13 probes) (Fig. 7, Table S2). Using all three tested development methods, we found that the *Krt19* mRNA expression pattern was the strongest at the proliferating bottom of the villi, decreasing towards the tip, corroborating previously published bulk and single-cell RNA-sequencing analyses (Moor et al., 2018).

### Conclusions

In conclusion, we have demonstrated the utility of the ‘one probe fits all’ OneSABER platform for whole-mount preparations of homeostatic and regenerating *M. lignano* flatworms. We also developed and validated a new fixation, permeabilization and hybridization protocol for *M. lignano* adapted to the use of short target-binding sequences of SABER probes with long concatemers, in addition to faster and loss-resistant sample handling by liquid-exchange mini columns. Importantly, we demonstrated the utility of our ISH protocol and OneSABER operation principles in other commonly used animal research models and sample types, namely planarian *Schmidtea meditteranea* and mouse FFPE tissue sections. We believe that OneSABER is a robust and versatile toolkit for fine-tuned ISH experiments and broad research applications.

## MATERIALS AND METHODS

### *M. lignano* culturing and sample preparation for ISH

*M. lignano* wild-type NL12 worms were cultured under standard conditions at 20°C as previously described (Mouton et al., 2024). For the ISH experiments, 3- to 5-week-old sexually mature animals were selected and prepared for fixation according to the protocol detailed in supplementary Materials and Methods, Section S4.1. For regeneration conditions, the tails of worms of the same age were amputated below the ovaries, worms were left to regenerate for 24 h without food, and were then fixed as in supplementary Materials and Methods, Section S4.1.

### *Schmidtea mediterranea* culturing and sample preparation for ISH

A laboratory culture of an asexual line of the planarian *S. mediterranea* was maintained in plastic containers with culture medium made by dissolving 0.5 g/l of hw-Marinemix^®^ professional (Wiegandt) in Milli-Q water. The animals were kept in the dark at room temperature and fed once a week with veal liver. Prior to the experiments, the worms were starved for at least a week.

### Mouse work and FFPE sample preparation

Small intestine tissue was obtained from a 12-week-old female C57BL/6J mouse bred at our local breeding colony at the Central Animal Facility of the University Medical Center Groningen. The mice were housed in a temperature-controlled environment (20-22°C) with *ad libitum* access to standard rodent chow and water and a 12 h light/12 h dark cycle. Before termination, mice were anesthetized by intraperitoneal injection of Hypnorm (fentanyl/fluanisone; 1 ml/kg) and diazepam (10 mg/kg). Mice were terminated by cardiac puncture under additional isoflurane anesthesia. The small intestine was rapidly excised and flushed with PBS containing protease inhibitors (cOmplete, Roche) before fixing in 4% formalin (VWR) for 24 h before paraffin embedding. Sections (5 µm) were generated, mounted on positively charged (silane-coated) glass slides, air-dried and incubated at 37°C overnight prior to further processing. The animal experiment was performed in conformity with the Dutch law on the welfare of laboratory animals and the experimental protocol and procedures were approved by the national committee for animal experiments and the Animal Welfare Body of the University Medical Center Groningen (study number: 2115290-02-012).

### Probes, PER concatemerization and HCR amplifiers

Except for the 5′-peroxidase-conjugated secondary probes for TSA SABER FISH (Eurogentec) and commercial HCR amplifiers for SABER-HCR FISH (Molecular Instruments), all DNA oligonucleotides were ordered from Integrated DNA Technologies. All probe sequences were ordered in a 25 nmol 96-well standard desalting format to reduce costs. The sequences, applications and purification methods for each oligonucleotide are listed in Tables S1 and S2. Antisense ssDNA probes with three different 3′ PER initiator sequences (p27, p28 and p30) (Kishi et al., 2019) were designed, checked *in silico* for target specificity, concatemerized to 200-500 nt by PER *in vitro*, and purified as detailed in supplementary Materials and Methods, Section S1.

### *In situ* hybridization and signal detection in whole-mount samples of *M. lignano* and *S. mediterranea*

A detailed step-by-step protocol for *M. lignano*, including buffer recipes, used antibodies, fluorescent tyramides, and mounting of the animals for microscopy, is provided in the supplementary Materials and Methods, Sections S2-S4. Samples developed with colorimetric AP SABER ISH were mounted in 80% glycerol/PBStw, while FISH samples were mounted in VECTASHIELD Vibrance (Vector Laboratories).

For *S. mediterranea* whole-mount samples, fixation, permeabilization and bleaching were performed according to a previously published protocol (Gaetano and King, 2023). Afterwards, the samples were processed as for *M. lignano* specimens following the same steps and using the same buffers and reagents starting from the ‘Pre-hybridization and hybridization’ part of supplementary Materials and Methods, Section S4.

### *In situ* hybridization and signal detection in mouse FFPE small intestine sections

The sections were deparaffinized by submerging the slides in xylene twice for 5 min, followed by two 1 min 96-100% ethanol incubations. Excess ethanol was removed from the sides of the slides by mopping with lint-free paper tissue before the slides were left to air-dry for 3-6 min at room temperature. Potential endogenous AP and peroxidase activities were quenched by evenly covering the section with BLOXALL^®^ Endogenous Blocking Solution (Vector Laboratories) and incubating at room temperature for 10 min, followed by two rinses in Milli-Q^®^ water. The slides were then mounted onto Sequenza^®^ Slide Racks where they remained until the end of the ISH signal development procedure. We found that the Sequenza rack mimics the use of liquid-exchange columns and allows easy, fast and low-volume solution exchange when working with tissue sections. Therefore, all the following procedures are performed using the rack. The slides were washed three times with 1 ml of 1× PBS, 0.1% Tween and once with 1 ml of Tris-EDTA buffer (pH 8). Distilled water was added at the bottom of the rack, and the top of the slide chambers was covered with cut-out fingertips of laboratory latex gloves to prevent evaporation. The rack was covered with an accompanying lid and placed in a pre-warmed hybridization oven at 70°C for 1 h. The rack was removed from the oven and allowed to reach room temperature for 10 min, followed by two 1 ml washes with 1× PBS, 0.1% Tween. Then, 500 µl of 20 µg/ml proteinase K (Jena Bioscience) were added and the slides were incubated 15 min at 37°C followed by two quick washes at room temperature with 1 ml of 1× PBS, 0.1% Tween. The rest of the procedures were followed as in the *M. lignano* protocol, starting from the ‘Pre-hybridization and hybridization’ part of supplementary Materials and Methods, Section S4 until Section S4.5 ‘Mounting on slides’. The wash buffer volumes were 500-1000 µl, minimum added volumes were 250 µl (hybridization buffers, antibodies, and signal development solutions). After the signal development procedures and washing, the slides were removed from the rack, briefly rinsed in Milli-Q^®^ water, stained with DAPI, rinsed again in Milli-Q^®^ water, and mounted in VECTASHIELD Vibrance (Vector Laboratories).

### Microscopy and image analysis

Widefield microscopy photos were taken using a Zeiss Axio Zoom V.16 stereo fluorescent microscope equipped with a PlanNeoFluar Z 2.3×/0.57 objective, HXP 200W lamp, and Zeiss filter sets 96HE (‘blue’; BP390/40, BP450/40), 38HE (‘green’; BP470/40, BP525/50), 43HE (‘red’; BP550/25, BP605/70) and filter set 50 (‘far-red’; BP640/30, BP690/50). Colorimetric ISH images were taken with an AxioCam MRc5 color camera, and fluorescent images were captured with an AxioCam HRm CCD camera. Confocal microscopy images were obtained using a Leica SP8x equipped with a HC PL APO CS2 63×/1.4 oil objective at the UMCG Imaging and Microscopy Center (UMIC). Raw microscopy images were processed using Fiji ImageJ software (Schindelin et al., 2012). The final figure panels were assembled in Inkscape v.1.3.2 (https://inkscape.org).

Measurements of fluorescent signal over background intensity ratios for Fig. 5B were performed using Fiji ImageJ using the following procedure. Full mounted worms (*n*=3 per signal development condition) were outlined/selected from the background using the wand (tracing) tool in the autofluorescence (no ISH signal) green channel. In the signal (far-red) channel, the mean gray intensity values of the worm [Mean worm fluorescence] and the non-worm background [Background fluorescence] areas were measured. To calculate signal over background ratios, [Mean worm fluorescence] was divided by [Background fluorescence].

## Supplementary Material



10.1242/develop.204775_sup1 Supplementary information
