# Supplementary material for: One probe fits all: a highly customizable modular RNA in situ hybridization platform expanding the application of SABER DNA probes
Source: Development. 2025 Jun 2;152(11):dev204775. doi: 10.1242/dev.204775 (PMC12148028; doi:10.1242/dev.204775)
Supplement: Supplementary file 1 [file develop-152-204775_review_history.pdf]

# One probe fits all: a highly customizable modular RNA in situ hybridization platform expanding the application of SABER DNA probes

Kirill Ustyantsev, Mattia Stranges, Filippo G. Volpe, Jan Freark de Boer, Folkert Kuipers, Stijn Mouton and Eugene Berezikov

DOI: 10.1242/dev.204775

**Editor**: James Briscoe

**Review timeline**

| | |
|---|---|
| Submission to Review Commons: | 25 May 2024 |
| Submission to Development: | 7 March 2025 |
| Editorial Decision: | 23 April 2025 |
| First revision received: | 8 May 2025 |
| Accepted: | 8 May 2025 |


---

Reviewer 1:

**Evidence, reproducibility and clarity**

Authors developed a customizable PER reaction system that is able to switch between different imager probes, as well as imaging modalities (Hapten, HCR, etc). This work will be of interest to biologists looking to validate gene expression, as well as biotechnologist looking to advance imaging-based spatial transcriptomics. The paper is well written and easy to read. The protocol is also very clear and well written. However, it is unclear how the method can enable new biological discovery.

Lack of demonstration of the applicability across sample types. Can the authors show some results in mammalian cells or tissues?

Fig.1 seems to suggest that the protocol for in vitro swapping of 3' concatemers happens in two consecutive PCR steps. I recommend indicating in the figure that the switching can be conducted in a single in vitro reaction.

Is it possible to multiplex the switching in one single reaction? For example, perform p27 to p28 and p29 to p30 simultaneously? This will be crucial for the split-probe methodology.

Did the authors encounter any switching hairpins sequence that does not work? If not, can they postulate, what are the requirements for the design of switching sequences.

Is there cross hybridization between the switched and original hairpins? For example, can the authors show that the signals from p27 and p30 do not overlaps?

Can the authors quantify results from the direct, AP, TSA, and HCR? What do you mean by 'narrow anatomical structures like neural chords (syt11) or muscles (tnnt2) seem less visible'?

**Referees cross-commenting**

© 2025. Published by The Company of Biologists under the terms of the Creative Commons Attribution License (https://creativecommons.org/licenses/by/4.0/).

I agree with reviewer #2 regarding the lack of comparison to standard SABER.

**Significance**

Authors developed a customizable PER reaction system that is able to switch between different imager probes, as well as imaging modalities (Hapten, HCR, etc). This work will be of interest to biologists looking to validate gene expression, as well as biotechnologist looking to advance imaging-based spatial transcriptomics. The paper is well written and easy to read. The protocol is also very clear and well written. However, it is unclear how the method can enable new biological discovery.

Reviewer 2:

**Evidence, reproducibility and clarity**

In their manuscript entitled "One probe fits all: a highly customizable modular RNA in situ hybridization platform expanding the application of SABER DNA probes" Ustyantsev et al. present combinations of the SABER (signal amplification by exchange reaction) method for RNA in situ hybridization (ISH) experiments with additional fluorescence amplification strategies such as alkaline phosphatase (AP) colorimetric-based, tyramide signal amplification-based (TSA) and hybridization chain reaction-based (HCR) ISH. All experiments are performed within whole-mount samples of M. lignano and single-plex data for a total of 7 genes and multiplexed data for up to three genes are shown. Based on an initial set of SABER probes, the OneSABER platform, standard SABER fluorescently-labeled readout oligos (imagers) can be easily replaced by oligos introducing the above mentioned alternative amplification strategies. Furthermore, the authors claim to have optimized existing sample protocols for in situ hybridization in M. lignano.

**Major comments:**

Overall, the study is carefully conducted and many of the author's claims are supported by data presented in their manuscript.

Please find my comments below:

- This work is building on standard SABER (a set of PER-extended primary probes that serve as landing pads for secondary fluorescently-labeled readout oligos) and pSABER (the readout oligo carries HRP instead of a dye for downstream TSA). The novelty of the work presented here is introducing additional variations of signal amplification, i.e. by using an hapten-labeled oligo to recruit a tertiary readout probe (antibodies conjugated with HRP or AP) or using SABER in combination with HCR. Since SABER can be seen as the underlying platform and pSABER was (arguably) also already introduced as a new platform by Attar et al. 2023, it seems difficult to introduce OneSABER as yet another new platform, of which standard SABER and pSABER are a part of. The reviewer encourages the authors to overthink the conceptual introduction, which in view of its certainly distinct novel features might allow a clearer distinction to previous work.
- Although the authors take care in tributing prior work, some of the studies are only mentioned in the results section, one of such cases is pSABER by Attar et al. 2023. The close relation between pSABER and SABER TSA (HRP on readout oligo vs. hapten on readout oligo + HRP-conjugated antibody) needs to be better positioned in the introduction, clearly framing earlier work, inspirations drawn etc.. This is in line with my previous point.
- Fig. 1 lists the individual modules of the OneSABER platform: i) standard SABER, ii) AP SABER, iii) SABER TSA, iv) pSABER (TSA FISH) (would recommend leaving it with original name when introducing it and include additional explanation in parentheses) and iv) SABER HCR. The main figures feature only AP SABER, SABER TSA and SABER HCR, for standard SABER and pSABER one must look up the SI. Since the authors describe the limited performance of standard SABER for one of their targets of interest (syt11) and since they have tested this target for all five conditions, it would be valuable to include a comparative view of all five platform modules in a single figure for syt11 or even also piwi, which also

© 2025. Published by The Company of Biologists under the terms of the Creative Commons Attribution License (https://creativecommons.org/licenses/by/4.0/). 

seems to have been tested for all five. Comparing the signal strength would be useful for the community, at least of each SABER variation compared to standard SABER.
- The description of how the authors designed their probes is very detailed and they also provide a nice step-by-step protocol for their individual commands using Oligominer and BLAT software. This reviewer is wondering how the authors chose their PER sequences that they appended to their mined set of homologous in situ hybridization probes (p27,p28,p30).
This is a general problem of multiplexed ISH approaches with single-stranded overhang, could the author's comment on potential self-interaction of the appended sequence with the homologous part, which might limit the PER efficiency, or elaborate on their choice?
- Fig.1 and l. 125 describe straightforward in vitro switching of the concatemer sequence for an existing set of primary probes as a central feature of the OneSABER platform. However, the authors to my knowledge do not show such experiments themselves and only cite the original SABER paper by Kishi et al. 2019. This reviewer would be grateful to be pointed toward where in Kishi et al. 2019 this was demonstrated, however in view of this central part of the swopping scheme in the OneSABER platform an experiment showing this swopping is missing.
- the description of Table S6 could use additional information in the legend such that the reader does not have to scroll down to Section S1 to retrieve the information (PER reaction, gel conditions, ladder is dsDNA, what are the individual bands)
- The manuscript features an extensive set of resources in main body, supplementary materials and protocols. It is important and usually not merited sufficiently making the effort to compare orthogonal approaches for a given aim. This reviewer particularly appreciates the detailed strengths & weaknesses discussion in Table S6.

**Minor comments:**

- Definitions should be consistent, in Fig. 1 all approaches are defined with FISH added, but this definition is not followed consistently in the main text.

**Optional:**

- The authors describe several newly developed optimization steps during sample preparation for M. lignano ISH experiments compared to established ones. If the data exists, they include a supplementary figure showing improvements of optimized protocol steps

**Referees cross-commenting**

I agree with most points raised by the other reviewers, especially with the lacking demonstration and related questions regarding swapping also raised by reviewer 1 and the questioned claim of translatability of OneSABER to other whole mount systems.

I do not question the value of this work in view of enabling new biological discovery, since it might accelerate/improve optimizations for RNA ISH experiments. In line with my comments, the manuscript would strongly benefit from a comparison to standard SABER demonstrating its insufficient signal for robust target detection.

**Significance**

Without a doubt this method-development focused study conducted by Ustyantsev et al. is a valuable resource featuring extensive sample optimization, protocols and guidelines for RNA in situ hybridization studies in M. lignano and as such deserves publication after the points raised were addressed. The manuscript is of high interest to the M. lignano community, to researchers conducting in situ hybridization experiments in larger/challenging-to-access samples and also to other methods developers.

© 2025. Published by The Company of Biologists under the terms of the Creative Commons Attribution License (https://creativecommons.org/licenses/by/4.0/).

*Field of expertise:* DNA nanotechnology and DNA-based multiplexed fluorescence imaging in mammalian cell culture & tissues.

Reviewer 3:

**Evidence, reproducibility and clarity**

**Summary:**

The authors of this study feature a proof-of-concept implementation of OneSABER ISHplatform, that combines single, and multiplex colorimetric and fluorescent approaches inwhole-mount samples of M. lignano. This includes RNA ISH, multiplex TSA and HCR FISH.The approach is supposed to provide advantages that reduce sample loss and sampleprocessing time and cost while being applicable to whole-mount samples of one organism,M. lignano, a powerful model that is used to study tissue regeneration. One of the moreobvious advantages is the use of this tool as an alternative to antibody staining for specificproteins. However, despite claiming applicability of this approach to other whole-mountorganisms, no evidence was shown to support that claim. In addition, the advantage of usingthis approach over other ISH protocols to study tissue regeneration in particular had not been shown.

**Major comments:**

- Despite including a whole figure (Figure 1) featuring the operation scheme of theOneSABER platform, the figure as well as the associated text fall short with respect toclearly stating the advantage of the different aspects of the platform. Consider a clearer andmore thorough explanation of the different aspects of the platfrom.
- Related to the first comment: A more detailed description of the similarities and/ordifferences of this platform relative to similar applications such as the study by Hall et al,2024.
- The authors describe the probes used as short, synthetic DNA probes targeting short RNAtranscripts. Are these probes Oligopaints (Beliveau et al, 2015)? Why is that not more clearlystated in the text?
- Line 105, p5: The authors state that the number of probes depends on the target RNAlength and its expression strength. This data should be in the main text and described indetail since it is a major aspect of the platform design.
- Figure 2 showcases one of the most compelling data supporting the versatility of the platform. Can the signals in each panel be quantified and compared to 1. Published Ab staining? Is there a clear correlation in the intensity of the signals? 2. Between Vector Blueand NBT? 3. Chemical staining and FISH signals?

**Minor comments:**

- The whole mount images and signals are often diffuse, can they be visualized using a DICwhere the morphology of the organism is clearer?
- In order to support the claim that this is a universal approach for whole-mount staining, canthe authors show an example of applicability to C. elegans?

**Significance**

The work presented by the authors is promising in its versatility to single, and multiplex colorimetric and fluorescent approaches. In particular, multiplexing several targets showcases the strength of this approach. However, the versatility, applicability to other whole-mount studies and as a tool to study tissue regeneration in this model organism are not shown in the manuscript. Additional experiments will be necessary to support several of these claims.

© 2025. Published by The Company of Biologists under the terms of the Creative Commons Attribution License (https://creativecommons.org/licenses/by/4.0/).

**Author response to reviewers' comments**

**Manuscript number:** RC-2024-02526

**Corresponding author(s):** Kirill Ustyantsev, Eugene Berezikov

1. General Statements

We thank the reviewers for their thorough evaluation of this manuscript. We are pleased that overall, they found our work and results valuable for the scientific community. Based on their feedback, we performed additional experiments and made several changes to strengthen the manuscript and expand the target audience.

*All three reviewers pointed out that the manuscript lacked demonstration of OneSABER method applicability across sample types (i.e., its claimed versatility) and other whole-mount systems beyond the Macrostomum lignano flatworm.*

We now include an additional results section with accompanying figures (Figs. 6 and 7) that demonstrate the application of OneSABER in whole-mount samples of another flatworm, the planarian *Schmidtea mediterranea* (Fig. 6), which is much larger than *M. lignano*, and in formalin- fixed paraffin-embedded (FFPE) mouse small intestine tissue sections (Fig. 7). We believe that these additional experiments on different sample types demonstrate the versatility of the OneSABER approach.

Please note that two more authors, Jan Freark de Boer and Folkert Kuipers, have been added for their contribution to mouse FFPE sections.

*Furthermore, two reviewers asked for an additional main figure with a comparison of the signal strengths between the different OneSABER methods.*

We have addressed this comment by including an additional results section and its adjacent figure (Fig. 5), where we provide a comparison of fluorescent signals from the same probes and gene but different OneSABER development methods.

Additionally, to implement the revisions, we modified Fig. 1 and Supplementary Fig. 6 and broadened Supplementary Tables S1-S2, S4-S6.

2. Point-by-point description of the revisions

**Reviewer #1**

*1) "Fig.1 seems to suggest that the protocol for in vitro swapping of 3' concatemers happens in two consecutive PCR steps. I recommend indicating in the figure that the switching can be conducted in a single in vitro reaction."*

We have changed Fig. 1 to make this clearer.

*2) "Is it possible to multiplex the switching in one single reaction? For example, perform p27 to p28 and p29 to p30 simultaneously? This will be crucial for the split-probe methodology."*

We did not test it. This should be possible if there is no overlap between the 3' initiator sequences. However, it seems counterproductive as the elongation efficiencies of switching reactions from the 3' initiator sequences to another concatemer may vary (Supplementary Fig. S6). Running independent extension/switch reactions and performing equimolar mixing of purified extended probes could be a better solution.

© 2025. Published by The Company of Biologists under the terms of the Creative Commons Attribution License (https://creativecommons.org/licenses/by/4.0/).

*3) "Did the authors encounter any switching hairpins sequence that does not work? If not, can they postulate, what are the requirements for the design of switching sequences."*

The design criteria followed the requirements postulated in the original SABER article and its Supplementary Materials (Kishi et al 2019). All switching hairpins we tested in the pairs of the 3 used 3' initiator sequences (p27, p28 and p30) worked, but elongation efficiencies varied (see an example in Supplementary Fig. S6).

*4) "Is there cross hybridization between the switched and original hairpins? For example, can the authors show that the signals from p27 and p30 do not overlaps?"*

The in situ hybridization results with swapped primary probes are shown in Fig. 6B (multiplexed HCR in *S. mediterranea*). All probes were originally designed using a p27 PER initiator. We swapped Smed-vit-1 with p30 and Smedwi-1 with p28. We also updated Fig. S6, by adding the second section (B) showing the in vitro results after concatemer swapping, as well as hybridization specificity of the secondary imager probes.

*5) "Can the authors quantify results from the direct, AP, TSA, and HCR? What do you mean by 'narrow anatomical structures like neural chords (syt11) or muscles (tnnt2) seem less visible'?"*

*"I agree with reviewer #2 regarding the lack of comparison to standard SABER."*

A comparison of fluorescent signals from the same probes/genes but different OneSABER development methods is shown in Fig. 5.

We have rephrased the sentence for clarity. From "As a result, despite higher intracellular resolution, some narrow anatomical structures like neural chords (syt11) or muscles (tnnt2) seem less visible for the human eye after SABER HCR (Figs. 3, 4)." to "As a result, despite higher intracellular resolution, some fine anatomical structures like neural chords (syt11) or muscles (tnnt2) are less resolved by widefield fluorescence microscopy after SABER HCR FISH compared to SABER TSA FISH"

**Reviewer #2**

*1) "This work is building on standard SABER (a set of PER-extended primary probes that serve as landing pads for secondary fluorescently-labeled readout oligos) and pSABER (the readout oligo carries HRP instead of a dye for downstream TSA). The novelty of the work presented here is introducing additional variations of signal amplification, i.e. by using an hapten-labeled oligo to recruit a tertiary readout probe (antibodies conjugated with HRP or AP) or using SABER in combination with HCR. Since SABER can be seen as the underlying platform and pSABER was (arguably) also already introduced as a new platform by Attar et al. 2023, it seems difficult to introduce OneSABER as yet another new platform, of which standard SABER and pSABER are a part of. The reviewer encourages the authors to overthink the conceptual introduction, which in view of its certainly distinct novel features might allow a clearer distinction to previous work."*

We agree with the reviewer's comments. We have added additional information in the Introduction section to clarify the novelty and key distinct features of OneSABER that justify its separation from other SABER protocols.

*2) "Although the authors take care in tributing prior work, some of the studies are only mentioned in the results section, one of such cases is pSABER by Attar et al. 2023. The close relation between pSABER and SABER TSA (HRP on readout oligo vs. hapten on readout oligo + HRP- conjugated antibody) needs to be better positioned in the introduction, clearly framing earlier work, inspirations drawn etc.. This is in line with my previous point."*

© 2025. Published by The Company of Biologists under the terms of the Creative Commons Attribution License (https://creativecommons.org/licenses/by/4.0/).

The pSABER preprint article by Attar et al. 2023 (now published in a peer-reviewed journal as Attar et al. 2025) is now mentioned in the Introduction, and its inspirational impact on our research is clearly stated.

*3) "Fig. 1 lists the individual modules of the OneSABER platform: i) standard SABER, ii) AP SABER, iii) SABER TSA, iv) pSABER (TSA FISH) (would recommend leaving it with original name when introducing it and include additional explanation in parentheses) and iv) SABER HCR. The main figures feature only AP SABER, SABER TSA and SABER HCR, for standard SABER and pSABER one must look up the SI. Since the authors describe the limited performance of standard SABER for one of their targets of interest (syt11) and since they have tested this target for all five conditions, it would be valuable to include a comparative view of all five platform modules in a single figure for syt11 or even also piwi, which also seems to have been tested for all five. Comparing the signal strength would be useful for the community, at least of each SABER variation compared to standard SABER."*

We agree with the reviewer's comments. Except for pSABER, a comparison of fluorescence signals from the same probes/genes but different OneSABER development methods is shown in Fig. 5. To make the comparison as objective as possible, all FISH developments were re-done using available "far red" fluorophores, except for pSABER. Unfortunately, our directly labeled HRP oligonucleotides for pSABER lost their activity after a year of storage at +4$^{O}$C. These conjugated oligonucleotides are very expensive and, given their limited shelf life, we cannot justify ordering a new batch for this experiment. Therefore, we only have the data for pSABER *syt11* with FITC green tyramide, which is not comparable to "far red" fluorophore signals. This issue has also been discussed in the main text.

In addition, we have modified Fig. 1, as suggested.

*4) "The description of how the authors designed their probes is very detailed and they also provide a nice step-by-step protocol for their individual commands using Oligominer and BLAT software. This reviewer is wondering how the authors chose their PER sequences that they appended to their mined set of homologous in situ hybridization probes (p27,p28,p30). This is a general problem of multiplexed ISH approaches with single-stranded overhang, could the author's comment on potential self-interaction of the appended sequence with the homologous part, which might limit the PER efficiency, or elaborate on their choice?"*

As being ourselves novice to SABER when we started our work, we based our selection of the p27, p28, and p30 PER sequences on their multiple co-occurrences in previous publications (Amamoto et al. 2019, doi: 10.7554/eLife.51452; Saka et al. 2019, doi: 10.1038/s41587-019- 0207-y; Wang et al. 2020, doi: 10.1016/j.omtm.2020.10.003; Salinas-Saavedra et al. 2023, doi: 10.1016/j.celrep.2023.112687; and Attar et al. 2023, doi: 10.1101/2023.01.30.526264). We did not consider the potential interference between PER concatemers and homologous primary probe-binding sequences. However, as all PER concatemers were specifically designed to lack G nucleotides to keep them from self-annealing (Kishi et al. 2019, doi: 10.1038/s41592-019-0404- 0), we assumed that it would also reduce potential annealing to the homologous part of the probe.

*5) "Fig.1 and l. 125 describe straightforward in vitro switching of the concatemer sequence for an existing set of primary probes as a central feature of the OneSABER platform. However, the authors to my knowledge do not show such experiments themselves and only cite the original SABER paper by Kishi et al. 2019. This reviewer would be grateful to be pointed toward where in Kishi et al. 2019 this was demonstrated, however in view of this central part of the swopping scheme in the OneSABER platform an experiment showing this swopping is missing."*

In the article by Kishi et al. 2019, concatemer switching/swapping is termed as "primer remapping". We found this term confusing because it does not describe the essence of the reaction. The in situ hybridization results with swapped primary probes are shown in Fig. 6B (multiplexed HCR in *S. mediterranea*). All probes were originally designed using a p27 PER initiator. We swapped Smed-vit-1

© 2025. Published by The Company of Biologists under the terms of the Creative Commons Attribution License (https://creativecommons.org/licenses/by/4.0/). 

with p30 and Smewi-1 with p28. We also updated Fig. S6, by adding the second section (B) showing the in vitro results after concatemer swapping, as well as hybridization specificity of the secondary imager probes.

*6) “the description of Table S6 could use additional information in the legend such that the reader does not have to scroll down to Section S1 to retrieve the information (PER reaction, gel conditions, ladder is dsDNA, what are the individual bands)”*

Probably, the reviewer meant Fig. S6. We now wrote a more detailed caption for the figure and extended it with a second panel (B) to illustrate the results of 3’ concatemer swapping.

*7) “the manuscript features an extensive set of resources in main body, supplementary materials and protocols. It is important and usually not merited sufficiently making the effort to compare orthogonal approaches for a given aim. This reviewer particularly appreciates the detailed strengths & weaknesses discussion in Table S6.”*

We thank the reviewer for the appreciation of our work.

*8) “Minor comments:*

*- Definitions should be consistent, in Fig. 1 all approaches are defined with FISH added, but this definition is not followed consistently in the main text.”*

These definitions are now made consistent throughout the text.

*9) “Optional:*

*-The authors describe several newly developed optimization steps during sample preparation for M. lignano ISH experiments compared to established ones. If the data exists, they include a supplementary figure showing improvements of optimized protocol steps”*

As almost every step and the buffer recipes were different from the original ISH protocol by Pfister et al. (2007) because of the use of liquid-exchange columns, different probes, and development chemistry, we believe that a comparison would be excessive. We think that the key difference points are already substantially highlighted in the results section.

**Reviewer #3**

*1) “Despite including a whole figure (Figure 1) featuring the operation scheme of the OneSABER platform, the figure as well as the associated text fall short with respect to clearly stating the advantage of the different aspects of the platform. Consider a clearer and more thorough explanation of the different aspects of the platfrom.”*

Details on the advantages and disadvantages of using different OneSABER methods in terms of their experimental application and cost efficiency are described in Supplementary Tables S4-S6 of the submitted manuscript. However, we agree that the description in Fig. 1 was too concise and also did not refer to these tables. We have expanded the description in Fig. 1.

*2) “Related to the first comment: A more detailed description of the similarities and/or differences of this platform relative to similar applications such as the study by Hall et al, 2024”*

The mere point of mentioning the preprint of Hall et al. 2024 (now peer-reviewed, https://doi.org/10.1016/j.celrep.2024.114892) was to acknowledge that in *M. lignano* the HCR technology has been previously applied (although only once), while all other previously published

© 2025. Published by The Company of Biologists under the terms of the Creative Commons Attribution License (https://creativecommons.org/licenses/by/4.0/).

works on *M. lignano* utilized canonical antisense RNA probes colorimetric in situ hybridization. We have extensively mentioned the HCR approach and its working principles throughout the submitted manuscript.

*3) "The authors describe the probes used as short, synthetic DNA probes targeting short RNA transcripts. Are these probes Oligopaints (Beliveau et al, 2015)? Why is that not more clearly stated in the text?"*

Oligopaints use oligo libraries as a renewable source of FISH probes, and these libraries are amplified with fluorophore-conjugated PCR primers. We used synthetic DNA probes directly. In this sense, our probe sets are not oligopaints. However, we used the OligoMiner pipeline of Oligopaints for the design of the probes, and thus used the same tiling strategy as oligopaints. We believe that this has been explained in the manuscript. Please refer to comment 4 of Reviewer 2.

*4) "Line 105, p5: The authors state that the number of probes depends on the target RNA length and its expression strength. This data should be in the main text and described in detail since it is a major aspect of the platform design."*

We believe that this statement is common sense, as one cannot design more than 5x 30-50 bp probes for 200 nt transcripts, while for a 2000 bp mRNA, the theoretical limit is ~50 probes. Similarly, weakly expressed genes (regardless of their length) would require either more probes to reach the detection threshold or stronger amplification through choice of concatemer length and/or signal developing techniques. We have rephrased this sentence in the main text to reflect this.

*5) "Figure 2 showcases one of the most compelling data supporting the versatility of the platform. Can the signals in each panel be quantified and compared to 1. Published Ab staining? Is there a clear correlation in the intensity of the signals? 2. Between Vector Blue and NBT? 3. Chemical staining and FISH signals?"*

Since *M. lignano* is a relatively new model, there are no published antibody stainings for *M. lignano* genes used in this study. Furthermore, colorimetric precipitate methods are not quantitative but rather qualitative, because their signal strength is proportional to both the target RNA level and the development time; thus, signals from weakly expressed transcripts can be "boosted" simply by longer development. Therefore, a correct quantitative comparison with colorimetric methods, as requested by the reviewer, was not possible. However, with some corrections on fluorophore differences and animal-to-animal variability, it is possible to roughly compare peak saturation intensities for FISH methods if the experiments are designed for this aim. We performed these experiments, and a comparison of fluorescent signals from the same probes/genes but different OneSABER development methods is shown in Fig. 5.

*Minor comments:*

*6) "The whole mount images and signals are often diffuse, can they be visualized using a DIC where the morphology of the organism is clearer?"*

We are unsure which images appear to be diffused to the reviewer. The other reviewers have not pointed out similar issues. Perhaps the question resolves once full-resolution uncompressed images are uploaded.

*7) "In order to support the claim that this is a universal approach for whole-mount staining, can the authors show an example of applicability to C. elegans?"*

This is now addressed. We included two additional results sections with two accompanying figures (Figs. 6 and 7) that demonstrate OneSABER's application in whole-mount samples of a much larger than *M. lignano* model flatworm, the planarian *Schmidtea mediterranea* (Fig. 6), as well as in

© 2025. Published by The Company of Biologists under the terms of the Creative Commons Attribution License (https://creativecommons.org/licenses/by/4.0/).

formalin-fixed paraffin-embedded (FFPE) small intestine tissue sections of a mouse model (Fig. 7).

---

**Original submission**

First decision letter

MS ID#: dev.204775

MS TITLE: One probe fits all: a highly customizable modular RNA in situ hybridization platform expanding the application of SABER DNA probes

AUTHORS: Kirill Ustyantsev, Mattia Stranges, Filippo G. Volpe, Jan Freark de Boer, Folkert Kuipers, Stijn Mouton and Eugene Berezikov

Dear Dr Berezikov,

Thank you for sending your manuscript to Development through Review Commons.

I have now received all the referees reports on the above manuscript, and have reached a decision. The referees' comments are appended below, or you can access them online: please go to .

The overall evaluation is positive and we would like to publish a revised manuscript in Development, provided that the referees' comments can be satisfactorily addressed. As you will see the referees ask for a couple of clarifications and I would encourage you to address these. Please attend to the reviewers' comments in your revised manuscript and detail them in your point-by-point response. If you do not agree with any of their criticisms or suggestions explain clearly why this is so. If it would be helpful, you are welcome to contact us to discuss your revision in greater detail. Please send us a point-by-point response indicating your plans for addressing the referees' comments, and we will look over this and provide further guidance.

Reviewer 1: I thank the authors for answering my questions. I have a small question for the revised manuscript regarding the analysis presented on Fig. 5b.
line 472 is not clear: "...using the following corrected total fluorescence formula: Integrated Density (Worm Area) - (Area of selected Worm × Mean fluorescence of signal-free inner worm area [background readings])."
Did the authors calculate the area integrated (or sum over the area of) fluorescent intensity in the 'signal' region and subtract that value by the background, which is defined as the mean fluorescence intensity of a background region multiplied by the same area? Would this be biased by the size of the area chosen (direct SABER seems to have a much smaller 'signal' area size)? Or is the signal area defined by the AP SABER ISH?

Reviewer 2: COMMENTS ON TEXT
I appreciate the authors' efforts in addressing my comments and now fully support the publication of their manuscript in Development.

COMMENTS ON DISPLAY ITEMS
Including Fig. 5 directly comparing all four SABER variants on target syt11 and the corresponding results section strenghtened the manuscript.

Reviewer 3: Summary:

© 2025. Published by The Company of Biologists under the terms of the Creative Commons Attribution License (https://creativecommons.org/licenses/by/4.0/). 

The authors of this study feature a proof-of-concept implementation of OneSABER ISH platform, that combines single, and multiplex colorimetric and fluorescent approaches in whole-mount samples of M. lignano to visualize gene expression. This includes RNA ISH, multiplex TSA and HCR FISH. The approach provides advantages that reduce sample loss and sample processing time and cost while being applicable to whole-mount samples of one organism, M. lignano, a powerful model that is used to study tissue regeneration. One of the more obvious advantages is the use of this tool as an alternative to antibody staining for specific proteins. The authors now have supported their claim of the applicability of this approach to other whole-mount organisms to include whole-mount planarians and fixed mouse tissue.

Major comment:
The authors have shown a beautiful quantitative comparison of the sensitivity and resolution of different OneSABER methods for M. lignano in Figure5B. It is pretty impressive to see the differences in signal-to-background with each of the methods and could be used as a benchmark for others in the field who want to apply the technology. It is important to show similar relative signal over background quantitation for Figures 6A and 7A-C.

---

**First revision**

<u>Author response to reviewers' comments</u>

Dear Editor,

We are delighted to hear that our manuscript is suitable for publication in Development and that the reviewers were largely satisfied with the revision. We would like to thank you and the reviewers for your time, critical evaluation and appreciation of our work.

General comments:

We formatted the manuscript to comply with the Development guidelines. Of note, the graph on Fig. 5B was modified to show the exact data points instead of means and standard deviations as required in the guidelines.

Abstract was shortened to fit to the 180 words limit. We included Running Title and Summary Statement in the title page. Although the title length is currently 130 symbols and exceeds the 120 words limit, if possible, we would like to keep it as is, since there were no objections from the reviewers' side.

To answer the question of Reviewer 1, we slightly extended and clarified the text on fluorescent image quantification to avoid potential confusions [see below].

Point-by-point reply to the reviewers:

Comments from the Reviewers:

*"Reviewer 1: I thank the authors for answering my questions. I have a small question for the revised manuscript regarding the analysis presented on Fig. 5b.*

*line 472 is not clear: "...using the following corrected total fluorescence formula: Integrated Density (Worm Area) - (Area of selected Worm × Mean fluorescence of signal-free inner worm area [background readings])."*

© 2025. Published by The Company of Biologists under the terms of the Creative Commons Attribution License (https://creativecommons.org/licenses/by/4.0/). 

*Did the authors calculate the area integrated (or sum over the area of) fluorescent intensity in the 'signal' region and subtract that value by the background, which is defined as the mean fluorescence intensity of a background region multiplied by the same area? Would this be biased by the size of the area chosen (direct SABER seems to have a much smaller 'signal' area size)? Or is the signal area defined by the AP SABER ISH?"*

We thank the reviewer for pointing out the issue with the formula provided and the confusion it created. In ImageJ, [Integrated density] equals [Mean intensity of gray pixel values] × [Area of measurement]. To eliminate the impact of the area instead of the "subtraction" sign there should have been the "division" sign. The final formula is then simplified to: [Mean worm fluorescence] / [Mean non-worm background fluorescence]. The correct formula was used, but it was written with the typo which is now corrected.

Additionally, we tried our best to select worms of the same age and size to avoid their impact on signal intensity variation between different methods. The signal area is simply the outline of the worm in the picture relative to its surroundings.

To clarify this, we modified the Materials and Methods paragraph as follows: *"Measurements of fluorescent signal over background intensity ratios for Fig. 5B were performed using Fiji ImageJ using the following procedure. Full mounted worms (N = 3 per signal development condition) were outlined/selected from the background using the wand (tracing) tool in the autofluorescence (no ISH signal) green channel. In the signal (far- red) channel, the mean gray intensity values of the worm [Mean worm fluorescence] and the non-worm background [Background fluorescence] areas were measured. To calculate signal over background ratios, [Mean worm fluorescence] was divided to [Background fluorescence]."*

*"Reviewer 2: COMMENTS ON TEXT*

*I appreciate the authors' efforts in addressing my comments and now fully support the publication of their manuscript in Development.*

*COMMENTS ON DISPLAY ITEMS*

*Including Fig. 5 directly comparing all four SABER variants on target syt11 and the corresponding results section strenghtened the manuscript."*

We thank the reviewer for his appreciation and support.

*"Reviewer 3: Summary:*

*The authors of this study feature a proof-of-concept implementation of OneSABER ISH platform, that combines single, and multiplex colorimetric and fluorescent approaches in whole-mount samples of M. lignano to visualize gene expression. This includes RNA ISH, multiplex TSA and HCR FISH. The approach provides advantages that reduce sample loss and sample processing time and cost while being applicable to whole-mount samples of one organism, M. lignano, a powerful model that is used to study tissue regeneration. One of the more obvious advantages is the use of this tool as an alternative to antibody staining for specific proteins. The authors now have supported their claim of the applicability of this approach to other whole-mount organisms to include whole-mount planarians and fixed mouse tissue.*

*Major comment:*

*The authors have shown a beautiful quantitative comparison of the sensitivity and resolution of different OneSABER methods for M. lignano in Figure5B. It is pretty impressive to see the differences*

© 2025. Published by The Company of Biologists under the terms of the Creative Commons Attribution License (https://creativecommons.org/licenses/by/4.0/).

*in signal-to-background with each of the methods and could be used as a benchmark for others in the field who want to apply the technology. It is important to show similar relative signal over background quantitation for Figures 6A and 7A-C."*

We thank the reviewer for acknowledging our work and the improvements in the manuscript. The purpose of Figs 6-7 was to show the versatility of the platform regardless of the model system used, and as such, the generated images are not suitable for proper comparative quantification. To perform comprehensive quantification for Figures 6A and 7A-C as in Figure 5B we would need to make new experiments from scratch with standardized fluorophores and include direct SABER FISH. We believe this effort is not justified, since we do not agree with the reviewer that it is important to perform such measurements. This is because whole mount RNA ISH is not a quantitative method and the choice of the development technique should be primarily guided by available resources, degree of multiplexing, and gene expression strength.

---

Second decision letter

MS ID#: dev.204775R1

MS TITLE: One probe fits all: a highly customizable modular RNA in situ hybridization platform expanding the application of SABER DNA probes

AUTHORS: Kirill Ustyantsev, Mattia Stranges, Filippo G. Volpe, Jan Freark de Boer, Folkert Kuipers, Stijn Mouton and Eugene Berezikov

Dear Dr Berezikov,

I am happy to tell you that your manuscript has been accepted for publication in Development, pending our standard publication integrity checks

© 2025. Published by The Company of Biologists under the terms of the Creative Commons Attribution License (https://creativecommons.org/licenses/by/4.0/).
